# Autoimmune Thyroiditis with Hypothyroidism Induced by Sugar Substitutes

**DOI:** 10.7759/cureus.3268

**Published:** 2018-09-07

**Authors:** Issac Sachmechi, Amna Khalid, Saba Iqbal Awan, Zohra R Malik, Mohaddeseh Sharifzadeh

**Affiliations:** 1 Internal Medicine, Icahn School of Medicine at Mount Sinai/Queens Hospital Center, New York, USA; 2 Internal Medicine, Icahn School of Medicine, Mount Sinai/Queens Hospital Center, New York, USA; 3 Diabetes Center, Ichan School of Medicine, New York City, USA; 4 Internal Medicine, Icahn School of Medicine at Mount Sinai/Queens Hospital Center, New York City, USA; 5 Internal Medicine, Icahn School of Medicine at Mount Sinai/Queen Hospital Center, New York, USA

**Keywords:** hashimoto’s thyroiditis, sugar substitutes, autoimmune, formaldehyde, hypothyroidism

## Abstract

The use of sugar substitutes (artificial sweeteners or non-nutritive sweeteners) has increased dramatically in the past few decades. They have been used as a substitute for sucrose (table sugar) in various diet-related disorders. Their excessive use has been linked to hyperphagia and obesity-related disorders. Hashimoto’s thyroiditis (chronic autoimmune thyroiditis) is a disease that involves the immune-mediated destruction of the thyroid gland, gradually leading to its failure. Animal studies report that artificial sweeteners affect the immune system. Moreover, animal studies show that sucralose diminishes the thyroid axis activity. We are presenting the case of a 52-year-old female with autoimmune thyroiditis with hypothyroidism (Hashimoto’s thyroiditis) induced by an excessive intake of beverages containing non-nutritive sweeteners. She was ruled out for any other autoimmune disorder. The association between Hashimoto’s thyroiditis and the excessive consumption of sugar substitutes is shown by the quick return of thyroid stimulating hormone and antibody levels to normal after eliminating the use of sugar substitutes. Thus, it suggests that the sugar substitutes were the culprit in the development of Hashimoto’s thyroiditis in our patient.

## Introduction

Sugar substitutes are very low energy or zero energy substances that are used to replace sugar in the diet. They are mostly in the market as "sugar-free," "diet," or "no sugar" substances. The commonly used sugar substitutes include saccharin, aspartame, sucralose, acesulphame K, and neotame. They are now very commonly used in a wide variety of foods and beverages, including, but not limited to, soft drinks, yogurt, jam, and chewing gum. Sugar substitutes are much sweeter than sucrose [1]. Aspartame is 200 times sweeter than sucrose whereas sucralose (a synthetic product of sucrose) generates 600 times more sweetness as compared to sucrose. This is because of the replacement of three hydroxyl groups in sucrose with three chlorine groups in sucralose [2]. The use of these sugar substitutes has been continuously increasing in the United States [3]. The use of sugar substitutes use is more prevalent in females [4]. The incidence of autoimmune diseases has also been increasing over the last decades. Its incidence has been increasing more in the West and the North [5]. In animal studies, sugar substitutes are linked to obesity and various malignancies [6-9]. Moreover, animal studies also suggest that the use of sugar substitutes has been linked to autoimmune diseases as well [10]. This association between the use of sugar substitutes use and the incidence of autoimmune diseases can be extrapolated to humans as well because the pharmacokinetics of sucralose in rats resembles that of humans [11]. Here, we report the first case of autoimmune thyroiditis with hypothyroidism induced by sugar substitutes whereas the abstract of this case has already been presented (Poster: Sachmechi I, Hussain S. Autoimmune Thyroiditis with Hypothyroidism Induced by Sugar Substitutes. The American Association of Clinical Endocrinologists Annual Congress; 2013, https://www.aace.com/files/abstracts-2013.pdf).

## Case presentation

A 52-year-old female with a history of consuming a high dose of artificial sweeteners was diagnosed with Hashimoto’s hypothyroidism. She had been using artificial sweeteners on an average of 6g/dl for 14 years. On presentation, her thyroid stimulating hormone (TSH) was 12.2 mIU/L (normal: 0.4-4.5), free T4 0.5 ng/dl (normal: 0.58-1.64), and anti-thyroid peroxidase antibody (Anti TPO Ab) 196 IU/ml (normal: <35). Treatment with levothyroxine 0.75 mg/day normalized her TSH, which remained between 1.23 mIU/L and 2.16 mIU/L during the following three years. She was also ruled out for other autoimmune disorders (Grave's disease, De Quervain thyroiditis) as well as drug-induced thyroiditis. The patient noticed a significant weight gain of 20 lbs since she started using artificial sweeteners. She correlated her weight gain with the use of artificial sweeteners, so she reduced and eventually stopped taking the sweeteners. Stopping the artificial sweeteners was followed by an unanticipated drop in her TSH to 0.005 mIU/L. The TSH remained suppressed despite the reduction in levothyroxine dose to 0.5 mg/day. After the complete discontinuation of levothyroxine, normal TSH and Anti-TPO Ab <20 IU/ml (normal: <35), thyroid stimulating immunoglobulin (TSI) 113% (normal less than 140%), and thyrotropin binding inhibiting immunoglobulin (TBII) <6.0% (normal: <16%) were achieved. She remained clinically euthyroid without any treatment during the subsequent follow-up visits. All the relevant lab values have been summarized below (Table 1).

**Table 1:** Relevant lab values TSH: Thyroid stimulating hormone; Anti-TPO: Anti-thyroid peroxidase antibody; TSI: Thyroid stimulating immunoglobulin; TBII: Thyrotropin binding inhibiting immunoglobulin

Laboratory parameter	Initial values	After treatment with levothyroxine	After discontinuation of sugar substitutes	After discontinuation of sugar substitutes and levothyroxine	Reference range
TSH (mIU/L)	12.2	1.23- 2.16	0.005	Normal	0.4-4.5
Free-T4 (ng/dl)	0.5	N/A	N/A	N/A	0.58-1.64
Anti-TPO (IU/ml)	196	N/A	N/A	<20	<35
TSI (%)	N/A	N/A	N/A	113	<140
TBII (%)	N/A	N/A	N/A	<6.0	<16

## Discussion

Hashimoto’s thyroiditis, also called chronic autoimmune thyroiditis, is a disease characterized by the gradual failure of the thyroid gland due to an immune-mediated destruction and apoptosis of the gland [12-14]. The two main types of Hashimoto thyroiditis include goitrous autoimmune thyroiditis and atrophic autoimmune thyroiditis. Both of these types have a common serological and pathological manifestation. These include lymphocytic infiltration and follicular destruction as well as high serum concentrations of antibodies to thyroid peroxidase (TPO) and thyroglobulin (TG) [15]. The causes include genetic and environmental factors [13-14]. Their association with sugar substitutes has not been studied in detail or reported earlier. The use of sugar substitutes (non-nutritive sweeteners or artificial sweeteners) has dramatically increased as a substitute for table sugar in people with diet-related disorders [16]. The most commonly used ones include aspartame, sucralose, and saccharin. The sugar substitutes are attributed with a large number of health-related side effects in animal studies, ranging from obesity to various malignancies [6-9]. Nonetheless, not much is known about the human implications of these findings, considering that the phenomenon of the excessive consumption of sugar substitutes is relatively new. 

According to studies, artificial sweeteners reduce the number of beneficial bacteria in the gut significantly, which leads to an increase in pH. As the gut microbes constitute around 80% of the immune system, this inhibits the immune system and thus the thyroid [6,10]. According to a study done on rats that compared the effects of sucrose on the thyroid with those of sucralose, sucralose diminishes the thyroid axis activity as opposed to sucrose, which stimulates it. Sucralose diminishes thyroid peroxidase activity, leading to a decrease in TSH, as well as in the plasma levels of T3 and T4 [17]. Aspartame is composed of two amino acids, phenylalanine and aspartame, which are connected to methanol [2]. Aspartame in the body further metabolizes to formaldehyde [18]. Moreover, a study done on male albino rats showed that formaldehyde (a metabolite of aspartame) causes the regression of the follicular epithelial cells of the thyroid gland, which leads to decreased levels of T3 and T4, and increased TSH levels. There is a possibility that, initially, formaldehyde increases the stimulation of the thyroid follicles, which rapidly worsens the synthetic capacity of the gland. This ultimately leads to the failure of the thyroid gland [19]. Formaldehyde, a metabolite of aspartame is reported to be associated with Type IV delayed hypersensitivity. Studies have shown that in the oral cavity of rats, mice, and humans, sucralose and sucrose stimulate the same sweet taste of the G-protein coupled receptor complex T1R2/T1R3 [20]. Moreover, the pharmacokinetics of sucralose is similar in humans and rats [11].

The patient we presented had been excessively taking beverages containing artificial sweeteners for many years and was diagnosed with Hashimoto's hypothyroidism. She recovered completely after she stopped taking the artificial sweeteners. The reason for this association between artificial sweeteners and Hashimoto's hypothyroidism could be the one mentioned above in the discussion or it may either be a rare idiosyncratic or a more generalized reaction to the high intake of artificial sweeteners. The long lag time between the use of artificial sweeteners and the clinical presentation of Hashimoto's thyroiditis might be a limiting factor, so large control studies should be done to confirm this association.

## Conclusions

This case emphasizes that in all patients diagnosed with Hashimoto's thyroiditis, the intake of sugar substitutes should be inquired into. If found positive, the intake should be discontinued and the thyroid function test should be followed up closely.

## References

[1] Grech A, Kam CO, Gemming L, Rangan A (2018). Diet-quality and socio-demographic factors associated with non-nutritive sweetener use in the Australian population. Nutrients.

[2] Yang Q (2010). Gain weight by “going diet?” Artificial sweeteners and the neurobiology of sugar cravings. Yale J Biol Med.

[3] Sylvetsky AC, Welsh JA, Brown RJ, Vos MB (2012). Low-calorie sweetener consumption is increasing in the United States. Am J Clin Nutr.

[4] Sylvetsky AC, Jin Y, Clark EJ, Welsh JA, Rother KI, Talegawkar SA (2017). Consumption of low-calorie sweeteners among children and adults in the United States. J Acad Nutr Diet.

[5] Lerner A, Jeremias P, Matthias T (2015). The world incidence and prevalence of autoimmune diseases is increasing. IJCD.

[6] Schiffman SS, Rother KI (2013). Sucralose, a synthetic organochlorine sweetener: overview of biological issues. J Toxicol Environ Health B Crit Rev.

[7] Fowler SP, Williams K, Resendez RG, Hunt KJ, Hazuda HP, Stern MP (2008). Fueling the obesity epidemic? Artificially sweetened beverage use and long-term weight gain. Obesity.

[8] Laska MN, Murray DM, Lytle LA, Harnack LJ (2012). Longitudinal associations between key dietary behaviors and weight gain over time: transitions through the adolescent years. Obesity.

[9] Rycerz K, Jaworska-Adamu JE (2013). Effects of aspartame metabolites on astrocytes and neurons. Folia Neuropathol.

[10] Mori K, Nakagawa Y, Ozaki H (2012). Does the gut microbiota trigger Hashimoto's thyroiditis?. Discov Med.

[11] Roberts A, Renwick AG, Sims J, Snodin DJ (2000). Sucralose metabolism and pharmacokinetics in man. Food Chem Toxicol.

[12] Hollowell JG, Staehling NW, Flanders WD, Hannon WH, Gunter EW, Spencer CA, Braverman LE (2002). Serum TSH, T(4), and thyroid antibodies in the United States population (1988 to 1994): national health and nutrition examination survey (NHANES III). J Clin Endocrinol Metab.

[13] Tamai H, Ohsako N, Takeno K (1980). Changes in thyroid function in euthyroid subjects with a family history of Graves' disease: a follow-up study of 69 patients. J Clin Endocrinol Metab.

[14] Kraiem Z, Baron E, Kahana L, Sadeh O, Sheinfeld M (1992). Changes in stimulating and blocking TSH receptor antibodies in a patient undergoing three cycles of transition from hypo to hyper-thyroidism and back to hypothyroidism. Clin Endocrinol (Oxf).

[15] Zimmerman RS, Brennan MD, McConahey WM, Goellner JR, Gharib H (1986). Hashimoto's thyroiditis. an uncommon cause of painful thyroid unresponsive to corticosteroid therapy. Ann Intern Med.

[16] Shankar P, Ahuja S, Sriram K (2013). Non-nutritive sweeteners: review and update. Nutrition.

[17] Pałkowska-Goździk E, Bigos A, Rosołowska-Huszcz D (2018). Type of sweet flavor carrier affects thyroid axis activity in male rats. Eur J Nutr.

[18] Trocho C, Pardo R, Rafecas I, Virgili J, Remesar X, Fernández-López JA, Alemany M (1998). Formaldehyde derived from dietary aspartame binds to tissue components in vivo. Life Sci.

[19] Patel KG, Bhatt HV, Choudhury AR (2003). Alteration in thyroid after formaldehyde (HCHO) treatment in rats. Ind Health.

[20] Bello NT, Hajnal A (2005). Male rats show an indifference-avoidance response for increasing concentrations of the artificial sweetener sucralose. Nutr Res.

